# Machine Learning Uncovers Novel Predictors of Peptide Receptor Radionuclide Therapy Eligibility in Neuroendocrine Neoplasms

**DOI:** 10.3390/cancers17172935

**Published:** 2025-09-08

**Authors:** Gábor Sipka, István Farkas, Annamária Bakos, Anikó Maráz, Zsófia Sára Mikó, Tamás Czékus, Mátyás Bukva, Szabolcs Urbán, László Pávics, Zsuzsanna Besenyi

**Affiliations:** 1 Department of Nuclear Medicine, University of Szeged, 6720 Szeged, Hungary; sipka.gabor@med.u-szeged.hu (G.S.); farkas.istvan@med.u-szeged.hu (I.F.); bakos.annamaria@med.u-szeged.hu (A.B.); miko.zsofia.sara@med.u-szeged.hu (Z.S.M.); czekus.tamas@janoskorhaz.hu (T.C.); urbansz@inf.u-szeged.hu (S.U.); pavics.laszlo@med.u-szeged.hu (L.P.); 2 Department of Oncotherapy, University of Szeged, 6720 Szeged, Hungary; maraz.aniko@med.u-szeged.hu; 3 Department of Immunology, Faculty of Science and Informatics, University of Szeged, 6720 Szeged, Hungary; bukva.matyas@brc.hu

**Keywords:** SPECT/CT, [^99m^Tc]Tc-EDDA/HYNIC-TOC SPECT/CT, neuroendocrine neoplasm, somatostatin receptor, machine learning, PRRT

## Abstract

*Background:* Neuroendocrine neoplasms (NENs) are a diverse group of malignancies in which somatostatin receptor expression can be crucial in guiding therapy. We aimed to evaluate the effectiveness of [^99m^Tc]Tc-EDDA/HYNIC-TOC SPECT/CT in differentiating neuroendocrine tumor histology, selecting candidates for radioligand therapy, and identifying correlations between somatostatin receptor expression and non-imaging parameters in metastatic NENs. *Methods:* This retrospective study included 65 patients (29 women, 36 men, mean age 61) with metastatic neuroendocrine neoplasms confirmed by histology, follow-up, or imaging, comprising 14 poorly differentiated carcinomas and 51 well-differentiated tumors. Somatostatin receptor SPECT/CT results were assessed visually and semiquantitatively, with mathematical models incorporating histological, oncological, immunohistochemical, and laboratory parameters, followed by biostatistical analysis. *Results:* Of 392 lesions evaluated, the majority were metastases in the liver, lymph nodes, and bones. Mathematical models estimated somatostatin receptor expression accurately (70–83%) based on clinical parameters alone. Key factors included tumor origin, oncological treatments, and the immunohistochemical marker CK7. Associations were found between age, grade, disease extent, and markers (CEA, CA19-9, AFP). *Conclusions:* Our findings suggest that [^99m^Tc]Tc-EDDA/HYNIC-TOC SPECT/CT effectively evaluates somatostatin receptor expression in NENs. Certain immunohistochemical and laboratory parameters, beyond recognized factors, show potential prognostic value, supporting individualized treatment strategies.

## Simple Summary

Neuroendocrine neoplasms (NENs) are a highly diverse group of tumors in which somatostatin receptor expression plays a central role in diagnosis and treatment planning. The present study underlines the value of [^99m^Tc]Tc-EDDA/HYNIC-TOC SPECT/CT in the assessment of receptor expression and the making of therapeutic decisions, particularly in the context of radioligand therapy. Using nonlinear mathematical modeling and imaging results, as well as analysis of histological, immunohistochemical, and laboratory data, we identified key factors—such as tumor origin, treatment history, CK7 status, and tumor markers—that can help predict receptor status even without imaging. These findings lend support to a more individualized approach to the treatment of metastatic NENs and may lead to new perspectives in imaging for this heterogeneous group of diseases.

## 1. Introduction

The incidence of neuroendocrine neoplasms (NENs), including neuroendocrine tumors (NETs) and neuroendocrine carcinomas (NECs), is steadily increasing [1,2]. Accurate histological differentiation of these malignancies, according to the WHO-2022 recommendation, is crucial, especially in view of their differentiation status. Well-differentiated forms are called neuroendocrine tumors, whereas poorly differentiated forms are called neuroendocrine carcinomas [3]. These usually asymptomatic tumors are typically diagnosed at an advanced stage, when distant metastases are already present [4]. Originating from various organs through the malignant transformation of neuroendocrine cells, these tumors display significant biological diversity. One of the hallmark features of neuroendocrine neoplasias is the expression of somatostatin receptors (SSTRs), which play a central role in both the pathogenesis and treatment of these tumors. SSTRs function as pivotal therapeutic targets for somatostatin analogues (SSA), pharmaceutical agents that can effectively inhibit hormone overproduction and delay tumor progression. Beyond their therapeutic relevance, SSTR expression levels also possess significant prognostic value. Numerous studies have demonstrated that higher SSTR expression correlates with improved therapeutic response and longer overall survival in NET patients [5]. Consequently, the SSTR expression profile not only serves as a fundamental basis for contemporary treatment strategies but also functions as a valuable biomarker, facilitating the prediction of patient outcomes and the development of personalized therapeutic approaches in neuroendocrine neoplasms. [6]. Both immunohistochemical and functional imaging techniques are available to detect this pattern. Imaging guidelines favor somatostatin receptor PET/CT because of its subtle resolution advantage, but the availability of the method is limited. However, SPECT/CT alternatives are widely available in nearly all countries around the world [7,8]. In instances where the test result indicates receptor positivity, peptide receptor radionuclide therapy (PRRT), a relatively novel yet extensively utilized selective nuclear medicine therapy, can be recommended as a treatment option in combination with conventional somatostatin analog therapy [9,10].

**Figure 1:**
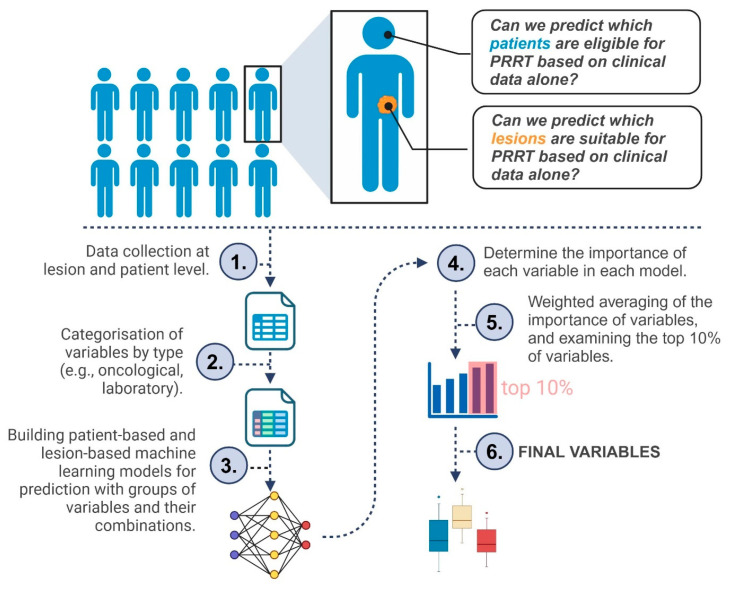
Simplified flowchart of the study. The figure illustrates the key steps of our analysis, from data collection through machine learning to finding the final variables.

Our study aimed to evaluate the effectiveness of [^99m^Tc]Tc-EDDA/HYNIC-TOC SPECT/CT in assessing somatostatin receptor expression across various NENs. We focused on its usefulness in differentiated neuroendocrine tumors and on identifying patients who would benefit from peptide receptor radioligand therapy. By analyzing receptor expression via SSTR SPECT/CT, we sought to understand variations in receptor density influenced by specific and non-specific histological, oncological, immunohistochemical, and laboratory parameters. Additionally, we examined correlations between receptor expression and non-imaging parameters in metastatic NENs, aiming to enhance understanding of tumor behavior and support more personalized treatment strategies. Figure 1 provides a concise flowchart of our study.

## 2. Materials and Methods

### 2.1. Patient Selection

This single-center retrospective study included 65 patients (29 women and 36 men; mean age 61 years; age range 25–84 years) with metastatic neuroendocrine neoplasms (neuroendocrine tumors and carcinomas). Only cases with histologically confirmed neuroendocrine neoplasia were enrolled. Exclusion criteria included the presence of another malignant primary tumor or the patient’s general condition being unsuitable or contraindicated for PRRT. In the patient-based analysis, researchers collected 100 binary and numeric features, while the lesion-based approach included 104 features. The most commonly used and newly identified variables were documented in detail throughout the data collection.

Immunohistochemical tests were carried out for chromogranin-A in all patients, and synaptophysin analysis was performed in most cases (83%). Several additional non-specific immunohistochemical markers were measured at relatively high frequencies, among which cytokeratin-7 (CK7; 51%), thyroid transcription factor 1 (TTF1; 43%), and cytokeratin-20 (CK20; 35%) were primarily employed to determine tumor origin. Among serum markers, chromogranin-A and neuron-specific enolase (NSE) were measured multiple times, while carcinoembryonic antigen (CEA), cancer antigen 19-9 (CA19-9), and alpha-fetoprotein (AFP) were also frequently tested prior to SPECT/CT (in 58%, 51%, and 32% of patients, respectively).

Somatostatin receptor scintigraphy and SPECT/CT scans relied on the liver and spleen as reference organs for the semiquantitative Krenning score. Lesions with radiopharmaceutical uptake higher than the liver were rated Krenning 3–4, indicating potential PRRT eligibility, while lesions with moderate uptake were labeled Krenning 2, and those with minimal or no uptake were designated Krenning 0–1. In the patient-based approach, only the least active lesion in each patient determined overall PRRT suitability.

Prior oncological treatments included surgical intervention or oncotherapy (chemotherapy or radiotherapy) in 45 out of 65 patients. Thirteen patients received somatostatin analog therapy, with an average withdrawal period of 36 days prior to the scheduled somatostatin receptor SPECT/CT scan.

### 2.2. Protocol for Somatostatin Receptor SPECT/CT Image Acquisition

All patients underwent somatostatin receptor scintigraphy. The radiopharmaceutical [^99m^Tc]Tc-EDDA/HYNIC-TOC SPECT/CT (Tektrotyd^©^, Polatom Ltd., Otwock, Poland; 700 MBq) was used for the study. An initial whole-body (AP/PA) scan was performed one hour after intravenous administration, though it was not included in the assessment. Four hours later, a short whole-body SPECT/CT scan (360°; 96 projections, 10 s/image; matrix: 128 × 128; pixel: 4.22 mm) was conducted, extending from the base of the skull to mid-thigh, and these images were analyzed in detail. SPECT data collection was followed by low-dose CT acquisition (120 kV and 70 mAs). All scans were performed on an integrated whole-body SPECT/CT system (Mediso AnyscanTRIO. Mediso Medical Imaging Systems Ltd., Budapest, Hungary). Occasionally, 24-hour SPECT scans were also taken for a more accurate assessment of certain lesions’ characteristics (10/65 patients); however, these scans were not evaluated in detail. Prior to imaging, patients were given an oral negative contrast agent (1000 mL of polyethylene glycol solution) to drink continuously, starting 60 min before the examination. This approach promoted bowel distention without introducing artefacts from contrast-material-induced attenuation correction.

### 2.3. Image Analysis and Lesion Detection

The somatostatin receptor scintigraphy was visually assessed by two nuclear medicine physicians, each with over 5 years of experience. In all cases, the 4-hour SPECT/CT scans were analyzed. A lesion was considered abnormal if there was focal and intense uptake of the radiopharmaceutical or if there was moderate or no tracer accumulation, but it was detectable on low-dose CT scans, with the presence of a malignant process confirmed by previous conventional radiological (CT, MRI, ultrasound, endosonography), endoscopic and nuclear medicine (bone scintigraphy, MIBG scintigraphy, FDG- or FDOPA-PET/CT) imaging modalities, or through histopathological examination during follow-up (see Supplement S1). The number of selected metastases per organ was maximized at 5. Metastatic lesions were primarily selected based on size, followed by activity level, allowing for the evaluation of lesions lacking somatostatin receptor expression. Findings in bones and lymph nodes were classified as part of the same organ group.

Somatostatin receptor SPECT/CT images were also analyzed semiquantitatively. Somatostatin receptor-positive lesions were identified semi-automatically using a minimum threshold of 42% [11]. Conversely, somatostatin-receptor-negative lesions were contoured based on CT morphology. The rate of accumulated activity in each lesion was calculated relative to reference organs (liver and spleen) (see Supplement S2), and a semiquantitative Krenning-score-based classification was performed [12]. Lesions with a low Krenning score (Krenning 0–2) exhibit reduced or similar radiotracer uptake compared to the liver. Conversely, malignant lesions with high scores (Krenning 3–4) demonstrate greater radiopharmaceutical uptake than the liver, potentially making them suitable candidates for radioligand therapy. The reference organ volume of interest (VOI) was always placed in a tumor-free segment, using a fixed 30 mm^3^ sphere. In one case, where splenectomy had been performed, a healthy kidney was used as the reference organ.

### 2.4. Data Analysis and Machine Learning Approach

The primary goal was to determine which clinical, pathological, and laboratory factors are most closely associated with SSTR expression in metastatic neuroendocrine neoplasms. We examined this question at two distinct levels. First, we focused on individual lesions to predict each one’s Krenning score and assess whether its activity might indicate suitability for peptide receptor radionuclide therapy. Second, we considered each patient as a whole, identifying the least active lesion and determining whether it would still qualify the patient for PRRT.

#### 2.4.1. Variable Grouping

To organize the extensive set of parameters, variables were grouped into four main categories:

1. oncological (group Onco, for example, previous treatments and tumor origin)
2. pathological (group Path, such as Ki-67 and tumor grade)
3. immunohistochemical (group Imm, including CK7 or CK20)
4. laboratory (group Lab, such as CEA, CA19-9, and AFP).

The imaging findings, including Krenning scores derived from SPECT/CT, were considered outcome data. The variable groups are presented in Supplement S3 and S4.

#### 2.4.2. Data Splitting and Model Development

To train and evaluate our models, we randomly split the dataset into an 80% training subset and a 20% test subset one hundred times, generating multiple partitions for more robust performance estimates.

During model development, we did not include all features simultaneously. Instead, models were trained using the predefined variable groups (Onco, Path, Imm, and Lab) both individually and in all possible combinations (e.g., Onco + Lab). This approach served a dual purpose: first, it allowed us to explore which clinical domains held the most predictive value; second, it helped mitigate the risk of overfitting given the relatively limited sample size compared to the number of available features.

We initially tested linear models as a baseline, but the principal focus was on Random Forest (RF) and Extreme Gradient Boosting (XGB), which are widely used decision-tree-based methods with proved efficiency in both classification and regression tasks. While RF builds multiple decision trees in the training phase and predicts using tree average prediction, XGB builds trees sequentially; therefore, it incrementally improves its prediction. Due to the methods’ loss functions, feature selection mechanism, and train phases, they can capture nonlinear relationships effectively [13,14,15,16]. Every classification algorithm was tested twice: once with the Synthetic Minority Oversampling Technique (SMOTE) applied exclusively to the training data, and once without SMOTE. Intuitively, SMOTE addresses class imbalances by generating new samples from the minority classes, thus creating a more homogeneous distribution [17,18]. Rather than simply duplicating original examples, SMOTE slightly varies the features of existing minority-class cases to produce synthetic instances. Because SMOTE is only applied to the training data, it does not inflate performance on the test set or leak information from the test set into the training process, preserving the validity of our performance estimates.

Model accuracy was assessed using the F1 score (harmonizing positive predictive value and sensitivity), and higher F1 values indicated superior overall classification performance [19].

#### 2.4.3. Feature Selection

**Figure 2:**
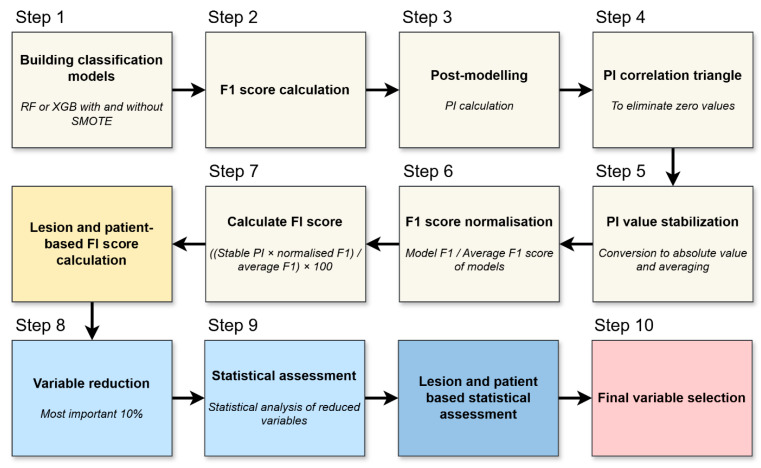
Flowchart of feature selection. The process shows step by step how to select the variables. (RF: Random forest, XGB: Extreme gradient boosting, PI: Permutation importance, FI: Feature importance).

We evaluated permutation-based feature importance per lesion and per patient to reveal the most influential variables in the models. This process is illustrated in Figure 2 and briefly outlined below.

The process begins by training Random Forest or XGBoost models on the data, sometimes including SMOTE as described previously (Figure 2, Step 1). Once each model is built, its predictive accuracy is assessed via the F1 score (Figure 2, Step 2).

Permutation importance (PI) was calculated to quantify the contribution of each variable to model performance (Figure 2, Step 3). For each feature, the values were randomly permuted 100 times across the dataset, while all other variables remained unchanged. The resulting decrease in the F1 score was averaged over the 100 permutations and used to estimate the variable’s relative importance [20].

Any features that exhibit negligible relevance—determined partly by examining correlations in the PI patterns—are removed (Figure 2, Step 4). This step ensures that the analysis focuses on factors that offer genuine explanatory power.

Because PI can fluctuate from run to run (i.e., across repeated model trainings using different data splits), the importance values were converted to absolute magnitudes and averaged (Figure 2, Step 5). Each model’s F1 score is normalized relative to the mean F1 of all models (Figure 2, Step 6). Good performers thus carry more weight, while weaker models’ results have proportionally less influence on subsequent calculations.

With stabilized PI and normalized F1 values in hand, a final feature importance (FI) is computed for both lesion- and patient-level analyses (Figure 2, Step 7). This yields two rankings—one focusing on individual metastatic lesions, the other on overall patient status.

Only the most influential variables—generally the top 10%—are retained for deeper consideration (Figure 2, Step 8). Eliminating lower-ranked features prevents the models from being burdened by weak predictors.

The refined list of variables is then subjected to classical statistical tests to verify that the same parameters singled out by the machine learning process also show statistically meaningful differences or associations (Figure 2, Step 9). This helps confirm their clinical relevance. Any variables confirmed at both the lesion and patient levels, and supported by standard statistical significance, are deemed most relevant (Figure 2, Step 10).

### 2.5. Additional Statistics

To corroborate the potential clinical utility of the highest-ranked predictors, we performed standard statistical tests on the final dataset. For continuous-type variables, such as lesion-to-liver and lesion-to-spleen uptake ratios, the Mann–Whitney U or Kruskal–Wallis test was employed, depending on whether two or more groups were compared. For binary outcomes, such as a patient’s likelihood of benefiting from PRRT, the Chi-square test was used. A *p*-value under 0.05 was considered indicative of statistical significance. All analyses were conducted using R version 4.3.3.

## 3. Results

### 3.1. Metastatic Neuroendocrine Neoplasms Frequently Exhibit PRRT-Suitable Lesions

Of the 65 patients, 51 presented with an advanced neuroendocrine tumor (NET), whereas 14 had neuroendocrine carcinoma (NEC). The most common primary site was the gastrointestinal system (GIS) in 33 patients (of whom 94% had an NET and 6% NEC), followed by the lung in 10 patients (equal 50–50% distribution of NET and NEC). In 7 patients, the primary tumor remained unknown, and 15 patients had tumors located in less frequent sites, such as the thyroid, reproductive organs, breast, MEN1–2 syndrome, or paraganglioma. Poorly differentiated cancers were more frequent in these less typical locations, with a ratio of 68% NET to 32% NEC. In terms of tumor grading, 24 were Grade I, 25 were Grade II, and 16 were Grade III. Consistent with an advanced-disease population, T3–4 lesions predominated in 32 cases, while 15 cases were considered T1–2 and 18 cases were considered Tx.

**Figure 3:**
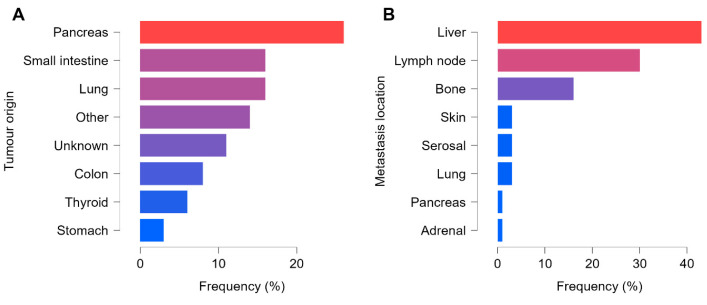
Distribution of tumor origin and metastasis location. The figure shows the distribution of tumors by origin (**A**) in the patient sample and the distribution of metastases by location (**B**). The blue-to-red gradient indicates increasing frequency.

In total, 392 pathological lesions were confirmed, comprising 47 primary tumors and 345 metastases. Every patient had at least one non-regional lymph node and/or distant metastasis. There was a slight predominance of vascular spread in 49 of the 65 cases, compared with 35 presenting lymphatic dissemination, and 24 patients had both distant and lymph node metastases detectable on SPECT/CT. Most of the metastatic lesions were detected in the liver (147 out of 392), followed by lymph nodes (105 out of 392) and bones (55 out of 392). The remaining 38 lesions occurred in the lungs, skin, adrenal glands, pancreas, or serosal surfaces. A total of 24 patients presented malignant involvement in three or more organs. The distribution of tumor origin and metastatic locations is summarized in Figure 3.

In gastrointestinal tumors, CK7 negativity was predominant, whereas in all other tumor types, CK7 positivity and negativity were observed at nearly the same rate (50–50%). Among all detected lesions, 197 were classified as Krenning 3–4, indicating radiotracer uptake above liver levels and rendering them potentially eligible for PRRT. Of the remaining lesions, 35 were Krenning 2 and 160 were Krenning 0–1. When considering only each patient’s least active lesion, 21 patients were categorized as PRRT-suitable, while 44 were not. Notably, among the 21 PRRT-eligible cases, one patient had a high-grade NET and one had NEC.

### 3.2. Model Performances: Complex Approaches Yield Better Accuracy

The modelling process began with linear analysis after grouping the features, but the results indicated low F1 values (mean F1 = 0.39), suggesting that there was no clear linear relationship between the study factors and somatostatin receptor expression. Consequently, further testing was linear models was discontinued.

#### 3.2.1. Lesion-Based Models Accurately Predict PRRT Suitability

For the lesion-based analysis, we ran six different models: two aimed to estimate the Krenning score, while the remaining four assessed the suitability for PRRT (ProPRRT). These calculations were performed using all 15 possible combinations of the four large variable groups. The models demonstrated the highest accuracy in predicting ProPRRT per lesion, with or without the SMOTE algorithm, achieving a mean F1 score of 0.83. Krenning score estimation was slightly lower but remained acceptable (F1 = 0.76). Among the groups, models incorporating pathological features performed the best, with a mean F1 of 0.83, while models lacking histological data scored 0.77. The most accurate predictor was an XGB-based (extreme gradient boosting) model estimating ProPRRT with the SMOTE algorithm using only pathological and laboratory data (F1 = 0.94).

#### 3.2.2. Patient-Based Models Benefit from Histological Data for Enhanced PRRT Prediction

**Figure 4:**
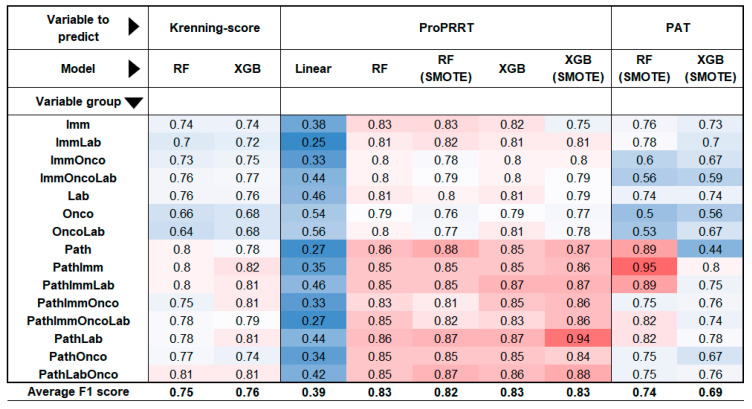
Heat map of the model performance. The heat map shows how efficiently each model predicted different output variables using each set of variables individually and in combination. The color of the cells changes from blue to red as the F1 score increases. (Imm: Immunohistochemical variables; Lab: Laboratory variables; Onco: Oncological variables; Path: Pathological variables; RF: Random Forest; XGB: Extreme Gradient Boosting; SMOTE: Synthetic Minority Oversampling Technique, PAT: Patient-based modelling).

In patient-based calculations, two models primarily assessed PRRT eligibility based on the patient’s least active lesion. Consistent with previous observations, models incorporating histological data proved to be successful predictors (mean F1 = 0.77), while those excluding histological factors showed a weaker performance (F1 = 0.63). Interestingly, random forest (RF) outperformed extreme gradient boosting (XGB) in this context, with F1 scores of 0.74 and 0.69. The best result in this group was achieved by an RF model using the SMOTE algorithm to estimate ProPRRT based on pathological and immunohistochemical features, reaching an F1 score of 0.95. Overall, mathematical models incorporating the pathological variables produced the highest F1 scores across evaluations. The results from the various models are summarized in Figure 4.

### 3.3. Feature Selection Identifies Key Predictive Biomarkers for PRRT Eligibility

The factors used in the different models can be ranked by their influence, measured by the permutation importance parameter. First, correlation triangles are created for both lesion- and patient-based descriptors to filter out factors with zero correlation (see in Supplement S5 and S6). To compare residual values across models with different strengths, standardization was applied, as shown in Figure 2. The resulting number, called feature importance, provides a basis for ranking factors in both lesion- and patient-based calculations.

In the lesion-based models, age emerged as the primary influencing factor, followed by CEA, metastasis location, serum chromogranin A, and CK7 marker. Similarly, in the patient-based ranking, age was observed as the primary factor, followed by serum chromogranin, number of lymph nodes involved, primary lung origin, and CA19-9 tumor marker. We identified the most relevant factors—those playing key roles in both simulations—allowing us to create a refined list of 12 factors for further testing with quantitative data during statistical assessment. The prominent factors identified in the simulated models include age, Ki-67, tumor origin (especially pancreatic and lung tumors), serum chromogranin A, tumor markers (CA19-9, CEA, AFP), immunohistochemical marker CK7, completed or ongoing oncological treatment, number of metastatic lymph nodes, and tumor size.

**Figure 5:**
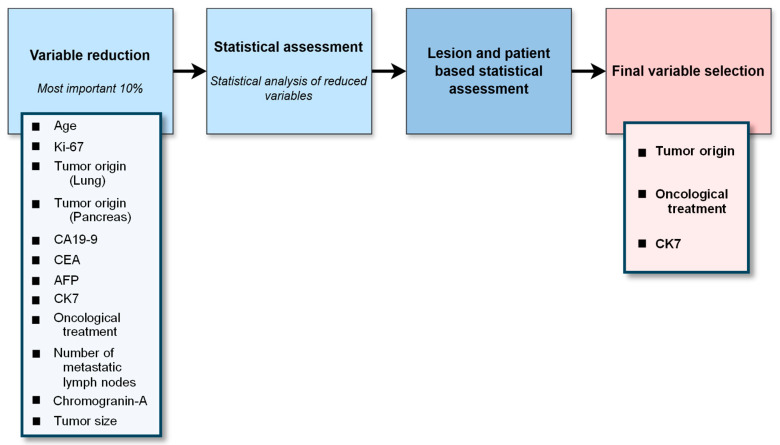
Feature selection. The flowchart illustrates the most significant variables that emerged following the model simulations, as well as those that remained after statistical assessment.

Factors were analyzed to determine whether the dataset extracted by a particular feature was significantly different from the remaining dataset or from the dataset defined by another factor. Mann–Whitney tests were used for numerical variables (Kruskal–Wallis for multiple groups), and Chi-square tests for binominal values. Among the parameters selected from previous models, both patient-based and lesion-based analyses revealed significant differences in somatostatin receptor expression based on tumor origin, previous or current oncological treatment, and CK7 status (Figure 5).

**Figure 6:**
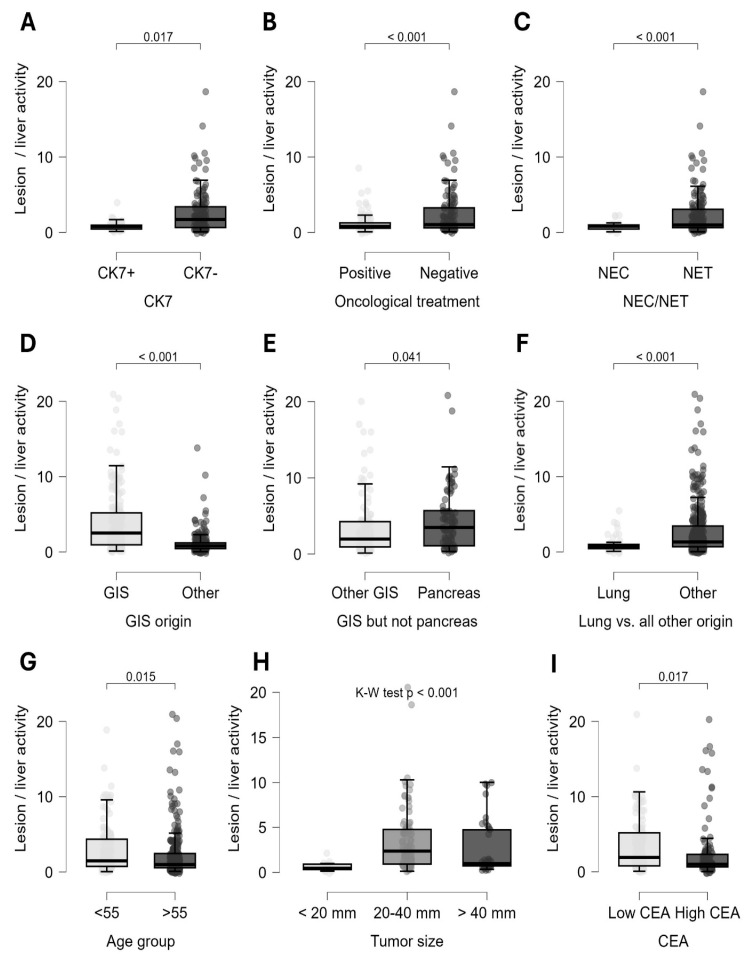
Lesion-based distribution of somatostatin receptor expression. This figure shows the lesion-based distribution of somatostatin receptor by CK7 factor (**A**), oncological therapy (**B**), degree of differentiation (**C**), different tumor origins (**D**–**F**), age (**G**), tumor size (**H**), and carcinoembryonic antigen (**I**).

Quantitative lesion-based analysis showed that neuroendocrine tumors (Mann–Whitney, *p* < 0.001), gastrointestinal neuroendocrine neoplasia (Mann–Whitney, *p* < 0.001), and, most notably, pancreatic origin (Mann–Whitney, *p* = 0.04) were associated with significantly increased somatostatin receptor expression. On the other hand, lung origin tumors (Mann–Whitney, *p* < 0.001) and neuroendocrine carcinoma (Mann–Whitney, *p* < 0.001) showed a notably lower radiopharmaceutical uptake. Adequate oncological treatment was associated with a significant reduction in SSTR expression (Mann–Whitney, *p* < 0.001). Interestingly, CK7-negative tumors exhibited a very strong association with increased somatostatin receptor status (Mann–Whitney, *p* < 0.001) (Figure 6).

**Figure 7:**
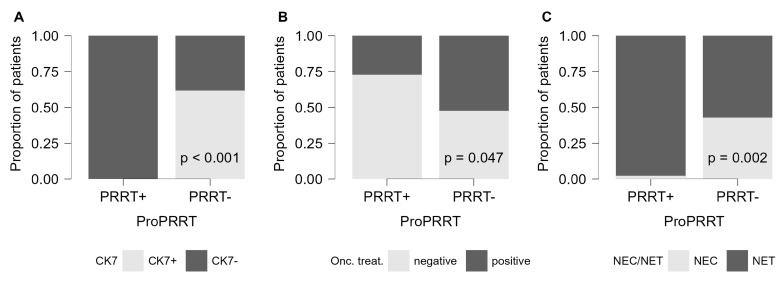
Patient-based results of PRRT suitability. Figure shows the patient-based results of PRRT suitability by CK7 factor (**A**), oncological therapy (**B**), and degree of differentiation (**C**).

In line with these findings, patient-based calculations showed that NETs (Chi-square, *p* = 0.002), gastrointestinal origin (Chi-square, *p* < 0.001), absence of oncological therapy (Chi-square, *p* = 0.047), and CK7 negativity (Chi-square, *p* < 0.001) were associated with the highest suitability for PRRT (Figure 7). Further lesion-level analysis of the CK7 marker showed that CK7 negativity was linked to significantly increased somatostatin receptor expression in several specific subgroups. This association was observed in tumors of unknown origin (Mann–Whitney, *p* = 0.003); in both neuroendocrine carcinomas (*p* = 0.001) and neuroendocrine tumors (*p* < 0.001); in grade I and II NETs (*p* = 0.005 and *p* = 0.011); and in lesions from patients undergoing oncological treatment (*p* = 0.017).

In lesion-based calculations, significant differences were observed between age groups above and below 55 years (Mann–Whitney, *p* = 0.015); tumor grade (Kruskal–Wallis, *p* < 0.001); tumor size (Kruskal–Wallis, *p* < 0.001); and tumor markers CEA (Mann–Whitney, *p* = 0.017), CA19-9 (Mann–Whitney, *p* = 0.005), and AFP (Mann–Whitney, *p* = 0.001) (Figure 5). Patients who were younger, had low-grade tumors, and showed normal CEA and CA19-9 but elevated AFP levels displayed increased radiopharmaceutical uptake in lesions on SPECT/CT scans. Among primary tumor sizes, lesions between 20 and 40 mm demonstrated the highest activity.

The influential effects of chromogranin-A and lymph node status were not confirmed during the statistical process. Additionally, although not being selected through modelling, patient-based calculations indicated that more widespread, multi-organ disease (Chi-square, *p* = 0.039) was associated with a reduced likelihood of PRRT eligibility. In lesion-based analyses, negativity for TTF1 (Mann–Whitney, *p* < 0.001) and CK20 (Mann–Whitney, *p* = 0.008) suggested increased somatostatin receptor expression.

## 4. Discussion

Both well-differentiated and poorly differentiated neuroendocrine neoplasms are invasive tumors that can metastasize to distant organs [3]. This makes it especially important to study a cohort of patients with distant metastases to identify potential therapeutic targets, such as somatostatin receptor expression. Although diagnostic guidelines recommend somatostatin receptor PET/CT for this purpose, we used a ^99m^Tc-labelled radiopharmaceutical ([^99m^Tc]Tc-EDDA/HYNIC-TOC SPECT/CT) in this study [21,22]. Our patient distribution and lesion prevalence by site align with literature findings, where gastrointestinal neuroendocrine neoplasias are the most common form, while rare tumor sites, such as the reproductive organs and breast, are predominantly neuroendocrine carcinomas [23,24,25,26].

While numerous publications on NETs form the basis of our current knowledge, NECs have become a distinct subgroup with aggressive clinical behavior and high mortality rates, especially under the new WHO-2022 classification [27,28]. Our study showed variability in somatostatin receptor expression on SPECT/CT between well-differentiated (neuroendocrine tumor) and poorly differentiated (neuroendocrine carcinoma) tumors, supporting the recommendations of international guidelines (Figure 6C and Figure 7C) [3,7].

Advancements in technology narrowed the resolution gap between PET/CT and SPECT/CT [29]. The primary hybrid diagnostic modality for neuroendocrine tumors is somatostatin-receptor-based imaging (SPECT/CT or PET/CT), while carcinomas fall within the spectrum of FDG-PET/CT [30]. With regard to SPECT/CT, ^99m^Tc -based imaging is superior to ^111^indium-based imaging due to its better diagnostic performance, as shown by Gabriel et al. [31,32,33,34]. For somatostatin receptor-based imaging, PET/CT generally offers superior sensitivity and specificity compared to SPECT. However, exceptions occur in both tumor types: some tumors may lack SSTR expression despite appearing as NETs, while others may express somatostatin receptors even when histologically identified as NECs. Exploring this gray area necessitates a comprehensive study of these tumors’ biological behavior. Our study aimed to identify and clarify links between specific factors to elucidate somatostatin receptor status across different types of malignant neuroendocrine lesions. These insights could potentially expand the current range of SSA-based therapies.

Currently, PRRT is indicated for inoperable, progressive, well-differentiated gastroenteropancreatic (GEP) neuroendocrine tumors that have been treated with somatostatin analogues. However, our findings suggest that PRRT might also offer a curative option for neuroendocrine neoplasms of non-gastrointestinal origins or higher grades [10,35,36]. Notably, combining PRRT with systemic chemotherapy may serve as an effective alternative in treating partially dedifferentiated neuroendocrine neoplasms, broadening the therapeutic options for these cases [37].

By utilizing RF and XGB-based modelling, as outlined in the Methods section, the objective was to identify nonlinear relationships between the various factors in this heterogeneous disease group [13,14,15,16]. This approach enabled a comprehensive exploration of the variables influencing the suitability of PRRT. Our advanced machine learning algorithms yielded high-quality results, showing that lesion-based models were more accurate predictors of key parameters compared to patient-based assessment. This improved accuracy was largely attributed to the higher data volume (392 vs. 65), which allowed for more robust insights in lesion-focused analyses. To address methodological differences among various machine learning techniques, we developed a standardized framework for determining feature importance across models. By aggregating over all subsets, we derived the overall feature importance. Through this approach, we identified 12 key parameters—age, Ki-67, pancreas origin, lung origin, serum chromogranin A, CA19-9, CEA, AFP, CK7 immunohistochemical factor, oncological treatment, number of metastatic lymph nodes, and tumor size— and ultimately validated three of these parameters explicitly using both patient- and lesion-based statistical methods: tumor origin, adequacy of oncological treatment and the immunohistochemical marker CK7 (Figure 5). Somatostatin receptors are well recognized for their overexpression in well-differentiated gastroenteropancreatic neuroendocrine neoplasms. Recent studies have demonstrated a progressive decline in somatostatin receptor 2A (SST2A) expression as malignancy advances from G1 neuroendocrine tumors to G3 neuroendocrine carcinoma [38,39]. Additionally, some data suggest variations in malignancy and overall survival rates based on the location of GEP-NEN along the gastrointestinal tract: malignant potential (Ki-67 level) is higher in hindgut tumors, resulting in lower survival rates compared to foregut tumors [40,41].

Our results partially align with previous findings: while GEP-NENs showed the strongest somatostatin receptor expression, tumors originating from the hindgut did not differ significantly from other gastrointestinal tumors. In contrast, pancreatic tumors had a significantly high somatostatin receptor expression. Lung-origin neuroendocrine neoplasms, though less frequently discussed, also demonstrated SSTR expression on the cell surface, albeit at levels lower than GEP-NETs—a pattern that our analysis similarly reflected (Figure 6D–F) [42,43].

**Figure 8:**
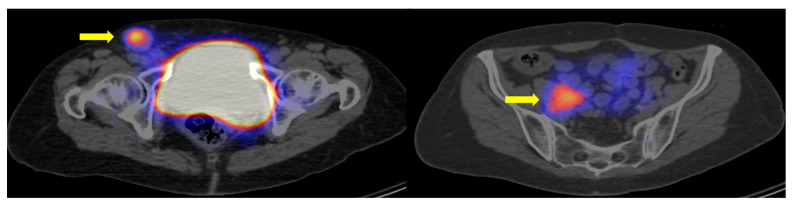
Somatostatin receptor expression in neuroendocrine carcinoma. A 73-year-old patient with grade III neuroendocrine carcinoma of unknown origin. (Ki67 = 50%) shown on [^99m^Tc]Tc-EDDA/HYNIC-TOC SPECT/CT axial section images. Immunohistochemical factors CK7 and TTF1 were negative. Arrows mark somatostatin receptor-positive lymph node metastases.

Our findings suggest that gastroenteropancreatic tumors, particularly those of pancreatic origin, are likely linked to increased radiopharmaceutical uptake, indicating a better prognosis. Conversely, lung tumors are likely to have reduced SSTR expression and a poorer prognosis [28,43]. It is worth noting that histologic dedifferentiation does not always result in a total loss of somatostatin receptors, making somatostatin-based imaging potentially valuable, especially for gastrointestinal neuroendocrine carcinomas (Figure 8).

Lesion-based analyses revealed a significantly increased uptake of radiopharmaceuticals in younger patients, in low-grade tumors, and when CEA and CA19-9 tumor markers levels were within the normal range (Figure 6G–I). Conversely, extensive multi-organ disease was linked to decreased uptake, which correlated with a higher likelihood of PRRT ineligibility. Factors such as grade, age, and disease progression are well-documented in the literature [44]. However, the relationship between tumor markers and somatostatin receptor expression has not been established in previous studies.

Our findings suggest an indirect link between certain tumor markers and increased radiopharmaceutical uptake, possibly reflecting tumor origin or stage. Normal tumor marker levels may indicate localized disease, whereas high values may reflect disseminated tumors. Additionally, CA19-9 is predominantly a marker for pancreatic tumors, while CEA is associated with colon, lung, and thyroid tumors [45,46,47,48]. This area holds promise for applications in clinical practice but will require further research to fully understand its implications.

**Figure 9:**
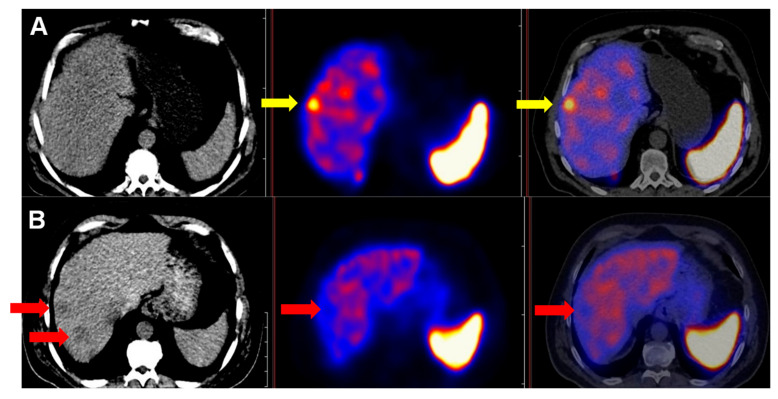
Demonstration of the clinical relevance of CK7 by an example. [^99m^Tc]Tc-EDDA/HYNIC-TOC SPECT/CT axial slice images showing a CK7 immunohistochemical marker-negative (**A**) and CK7-positive (**B**) patient with a Grade II neuroendocrine tumor of unknown origin. Arrows mark the metastatic lesions.

Among immunohistochemical factors, CK7 emerged as a dominant marker throughout the study. Overall, CK7 negativity was associated with increased SSTR expression in both patient- and lesion-based comparisons (Figure 6A; Figure 7A). Furthermore, our results confirmed CK7-negative and corresponding somatostatin receptor-positive status in challenging diagnostic subgroups, including tumors of unknown origin, post-chemo-radiotherapy patients, and high-grade neoplasia. Figure 9 illustrates the importance of CK7 through a specific case. These findings have not been previously reported in the literature.

An immunohistochemical analysis, albeit limited to a NEC population, has reached similar conclusions to ours, showing that tumors of gastrointestinal origin are predominantly CK7-negative, whereas those of lung origin are CK7-positive (*p* < 0.0001), and that GEP-NECs are significantly more likely to express somatostatin receptors (*p* = 0.0021) [44,47]. The analysis of other immunohistochemical factors, including CK20 and TTF1 negativity, further highlighted increased somatostatin receptor expression in lesions, primarily aiding in the distinction between gastrointestinal and pulmonary origins [49,50,51].

Oncological therapies aim to modify the biological behavior of tumors and inhibit their proliferation. Following such therapeutic protocols can mitigate the defining characteristics of the tumor type. Assessing therapy response through molecular hybrid imaging is already an integral part of standard practice in most cases [52]. In this context, our results indicated that even oncological therapies not specifically targeting somatostatin receptor expression can induce downregulation of these transmembrane proteins—a change that can be visualized through somatostatin receptor-based imaging (Figure 6B and Figure 7B).

## 5. Limitations

This is a single-center retrospective study, which limits the generalizability of these findings. Our method utilized ^99m^Tc-based somatostatin receptor SPECT/CT for its general availability and superior sensitivity compared to other SPECT radiopharmaceuticals, although its sensitivity remains below that of the internationally endorsed somatostatin receptor PET/CT. In daily clinical practice, our selected patient population with metastatic neuroendocrine neoplasia is highly heterogeneous, representing only those referred to our institute. Histopathological examination was not available for all lesions, so we relied on follow-up imaging studies to confirm all abnormalities as thoroughly as possible. Additionally, due to the retrospective nature of the data collection, not all immunohistochemical and laboratory parameters were determined for every patient.

## 6. Conclusions

Using machine learning algorithms, we identified factors influencing somatostatin receptor expression, including tumor origin, the effects of oncological therapy, and an unexplored factor: the immunohistochemical marker CK7. These findings underscore the pronounced heterogeneity of neuroendocrine neoplasms, suggesting that the precise assessment of somatostatin receptor status may depend on multiple variables. We hope that future research will expand the use of these less commonly studied immunohistochemical markers within the realm of hybrid imaging.

## Data Availability

Data are contained within the article or Supplementary Materials.

## Supplementary Materials

The following supporting information can be downloaded at: https://www.mdpi.com/article/10.3390/cancers17172935/s1, Supplement S1: Basic dataset; Supplement S2: Lesion based quantitative and semiquantitative parameters; Supplement S3: Lesion based features; Supplement S4: Patient based features; Supplement S5: Lesion based features’ permutation importance correlation triangle; Supplement S6: Patient based features’ permutation importance correlation triangle.

## Abbreviations

The following abbreviations are used in this manuscript

SPECT	Single photon emission computer tomography
PET	Positron emission tomography
CT	Computer tomography
MRI	Magnetic resonance imaging
PRRT	Peptide receptor radioligand therapy
NEN	Neuroendocrine neoplasm
NET	Neuroendocrine tumor
NEC	Neuroendocrine carcinoma
SSTR	Somatostatin receptor
SSA	Somatostatin analog
SST2A	Somatostatin receptor type 2A
GIS	Gastrointestinal system
GEP	Gastro-entero-pancreatic
CK7	Cytokeratin-7
CK20	Cytokeratin-20
TTF-1	Thyroid transcription factor 1
CEA	Carcinoembryonic antigen
CA19-9	Cancer antigen 19-9
AFP	Alpha-fetoprotein
NSE	Neuron-specific enolase
WHO	World Health Organization
MIBG	Metaiodobenzylguanidine
FDG	Fluorodeoxyglucose
F-DOPA	Fluoro-dihydroxy phenylalanine
VOI	Volume of interest
AP	Antero-posterior
PA	Postero-anterior
RF	Random forest
XGB	Extreme Gradient Boosting
SMOTE	Synthetic Minority Oversampling Technique
PI	Permutation importance
FI	Feature importance
