# Supplementary material for: Machine Learning Uncovers Novel Predictors of Peptide Receptor Radionuclide Therapy Eligibility in Neuroendocrine Neoplasms
Source: Cancers (Basel). 2025 Sep 8;17(17):2935. doi: 10.3390/cancers17172935 (PMC12428411; doi:10.3390/cancers17172935)

Supplement S1: *Basic dataset*

Number	Age	Gender	NET or NEC	Primary tumor location	Metastasis locations	Histology from primary	Histology from metastasis	CT	MRI	US	Endosonography or endoscopy	PET/CT or other nuclear medicine techniques	Follow up
1	53	Woman	NET	Pancreas	Liver	X	X		X	X			4 year 7 month
2	45	Man	NET	Thyroid	Liver, Lymph node, Bone, Lung	X		X		X			7 year 2 month
3	69	Man	NEC	Cervix	Lymph node	X		X	X			X (FDG)	1 year †
4	68	Woman	NET	Pancreas	Liver, Bone		X	X	X	X			6 year 1 month
5	70	Man	NET	Unknown	Liver, Lymph node	X	X	X				X (DOPA)	6 year
6	65	Man	NET	Prostate	Liver, Lymph node, Bone	X		X	X	X			1 year †
7	66	Man	NEC	Lung	Lymph node		X	X	X				6 year 1 month
8	41	Man	NET	Small Intestine	Liver	X		X		X	X		5 year 1 month
9	72	Man	NET	Small Intestine	Liver	X	X	X	X	X	X		2 year 5 month †
10	67	Woman	NET	Pancreas	Liver	X		X		X			5 year 6 month
11	25	Man	NET	Paraganglioma	Lymph node, Bone	X						X (MIBG)	9 month †
12	71	Woman	NET	Unknown	Liver		X	X					8 month †
13	73	Man	NET	Unknown	Lymph node, Serosal		X	X		X	X		3 year 6 month †
14	52	Man	NEC	Unknown	Liver		X	X		X			4 year 1 month †
15	45	Man	NET	Thyroid	Liver, Lymph node, Bone, Pancreas, Lung, Serosal	X	X	X			X		1 year 3 month †
16	69	Man	NET	Pancreas	Liver	X	X	X	X	X			3 year 11 month †
17	49	Man	NET	Pancreas	Liver, Bone	X	X	X	X				1 year 4 month †
18	64	Man	NET	Lung	Liver, Lymph node	X	X	X		X			4 year 6 month
19	63	Woman	NET	Small Intestine	Liver	X	X	X	X	X			4 year 2 month
20	82	Man	NET	Small Intestine	Liver	X		X	X				6 month †
21	60	Woman	NEC	Breast	Liver, Lymph node, Lung	X	X	X	X			X (FDG)	5 month †
22	60	Man	NEC	Thyroid	Lymph node, Skin	X	X	X	X				3 month †
23	44	Man	NET	Thymus (MEN-1)	Lymph node, Bone, Serosal	X	X	X	X	X			7 year
24	69	Man	NEC	Stomach	Lymph node	X	X	X	X		X		2 year 1 month †
25	39	Man	NET	Thyroid	Lymph node	X	X	X	X				6 year 10 month
26	58	Man	NET	Small Intestine	Liver, Bone	X	X	X		X		X (DOPA)	4 year 1 month
27	77	Man	NET	Colorectal	Lymph node	X		X					6 month †
28	38	Woman	NET	Thyroid (MEN-2A)	Bone	X	X	X				X (DOPA + Bone scan)	4 year 4 month
29	70	Woman	NET	Lung	Liver, Bone	X	X	X				X (Bone scan)	3 year 3 month †
30	59	Man	NET	Lung	Liver, Bone, Lung	X	X	X				X (Bone scan)	8 month †
31	51	Man	NET	Unknown	Liver, Lymph node	X			X		X		6 year 1 month
32	56	Woman	NET	Pancreas	Liver	X		X	X	X			5 year 2 month
33	65	Woman	NEC	Genital	Lymph node, Bone	X	X	X		X		X (FDG)	3 month †
34	66	Man	NET	Colorectal	Liver, Lymph node, Bone, Skin	X		X			X	X (Bone scan)	1 year 5 month†
35	71	Man	NEC	Lung	Liver, Bone		X	X	X			X (FDG)	5 year
36	66	Woman	NEC	Stomach	Serosal	X	X	X			X		3 month †
37	67	Woman	NEC	Lung	Lymph node, Adrenal		X	X				X (FDG)	10 month †
38	78	Man	NET	Small Intestine	Liver	X	X	X	X	X	X		2 year 2 month †
39	63	Woman	NET	Pancreas	Liver	X	X	X	X	X	X		3 year 8 month
40	54	Man	NET	Pancreas	Lymph node	X		X	X		X	X (Bone scan)	3 year 5 month
41	59	Woman	NET	Pancreas	Lymph node	X		X		X			1 year 2 month †
42	81	Man	NET	Small Intestine	Liver		X		X	X	X		3 year 3 month
43	38	Man	NET	Thyroid (MEN-2A)	Lymph node	X	X	X				X (DOPA)	3 year 3 month
44	66	Man	NET	Thyroid (MEN-2A)	Lymph node	X	X	X				X (DOPA)	3 year 3 month
45	53	Woman	NET	Pancreas	Liver		X	X	X				1 year 5 month †
46	79	Woman	NET	Colorectal	Liver, Lymph node	X		X	X	X	X	X (Bone scan)	2 year †
47	73	Woman	NEC	Unknown	Lymph node		X	X		X			1 year 2 month †
48	45	Woman	NET	Pancreas	Liver	X	X	X	X	X			3 year 1 month
49	73	Man	NET	Pancreas	Lymph node	X		X		X			3 year
50	78	Woman	NET	Pancreas	Liver		X	X	X				1 year 6 month †
51	68	Man	NET	Lung	Liver, Lymph node, Adrenal	X		X					1 year 8 month †
52	47	Woman	NET	Pancreas	Liver		X	X		X	X		8 month †
53	74	Woman	NET	Small Intestine	Liver, Lymph node, Serosal	X	X	X		X	X		2 year 9 month
54	66	Woman	NET	Lung	Serosal	X	X	X					2 year 9 month
55	60	Woman	NET	Small Intestine	Liver, Lymph node		X	X		X	X		2 year 8 month
56	67	Man	NET	Prostate	Liver, Lymph node, Bone	X		X					2 year 8 month
57	84	Woman	NET	Lung	Liver, Bone	X	X	X	X			X (FDG)	1 month †
58	66	Woman	NEC	Breast	Liver, Lymph node	X	X		X	X	X	X (FDG)	10 month †
59	58	Woman	NEC	Cervix	Lymph node	X		X	X	X		X (FDG)	2 year 7 month
60	38	Woman	NEC	Unknown	Liver, Lymph node, Bone		X	X	X			X (FDG)	6 month †
61	53	Man	NET	Pancreas	Liver, Lymph node	X		X	X	X			2 year 7 month
62	66	Man	NET	Small Intestine	Lymph node	X	X	X			X		2 year 7 month
63	57	Man	NET	Unknown	Lymph node, Serosal	X	X	X	X	X			2 year 3 month
64	65	Man	NET	Colorectal	Liver, Bone	X		X	X	X			2 year 3 month
65	65	Woman	NEC	Lung	Serosal	X	x	X	X			X (FDG)	1 year †

Supplement S2: ***Lesion based quantitative and semiquantitative parameters***

The table is attached in a separate Excel file.

Supplement S3: *Lesion based features*

Lesion based features			
Oncological	Pathological	Immunohisto-chemical	Laboratory
Age	NET	Chromogranin<3	CEA
Surgery	NEC	Chromogranin≥3	Chromogranin-A
Gender	Met. Lymph node	Synaptophysin<3	CA19
Chemoth.	Met. Liver	Synaptophysin≥3	AFP
Biol. Th.	Met. Pancreas	CD06<3	NSE
SSA absence time	Met. Cutan	CD56≥3	CA72-4
Octreotide	Met. Lung	Somatostatin rec+	CA125
Irradiation	Met. Bone	Somatostatin rec-	CA15-3
SSA th.	Met. Serosal	Calcitonin+	
Lanreotide	Met. Adrenal	Calcitonin-	
	Pancreas origin	TTF+	
	Colorectal origin	TTF-	
	Lung origin	Syntaxin<3	
	Small Int. origin	Syntaxin≥3	
	Thyroid origin	CK7+	
	Unknown origin	CK7-	
	MEN2 origin	CK20+	
	Prostata origin	CK20-	
	Met. Serosal	CDX2+	
	Genital origin	CDX2-	
	MEN1 origin	S100+	
	Cervix origin	S100-	
	Breast origin	p63+	
	Stomach origin	p63-	
	Adrenal origin	Vimentin+	
	Grade I	Vimentin-	
	Grade II	Cadherin+	
	Grade III	Cadherin-	
	Ki-67	SATB+	
	Mytosis	SATB-	
	Primer or Met.	GATA3+	
	T1	GATA-	
	T2	CKKL4-	
	T3	CKKL+	
	T4	MUC0AC+	
	Tx	MUC0AC-	
	Tumor size		
	N0		
	N1/2		
	Nx		
	Lymph node number		
	V0		
	V1		
	L0		
	L1		
	Pn0		
	Pn1		
	R0		
	R1		
	Rx		

Supplement S4: *Patient based features*

Patient based features			
Oncological	Pathological	Immunohisto-chemical	Laboratory
Age	NET	Chromogranin<3	Chromogranin-A
Gender	NEC	Chromogranin≥3	CEA
Chemotherapy	Pancreas origin	Synaptophysin<3	NSE
Irradiation	Thyroid origin	Synaptophysin≥3	CA19
Surgery	Cervix origin	CD06<3	CA72-4
Biol. Th.	Prostata origin	CD56≥3	AFP
Any oncol. Th.	Lung origin	Somatostatin rec+	CA125
SSA th.	Small intest. origin	Somatostatin rec-	CA15-3
SSA absence time	Adrenal origin	Calcitonin+	
Oktreotide	Breast origin	Calcitonin-	
Lanreotide	MEN1 origin	TTF+	
	MEN2 origin	TTF-	
	Stomach origin	Syntaxin<3	
	Colorectal origin	Syntaxin≥3	
	Genital origin	CK7+	
	Unknown origin	CK7-	
	Grade I.	CK20+	
	Grade II	CK20-	
	Grade III	CDX2+	
	Ki-67	CDX2-	
	Primary inside?	S100+	
	T1	S100-	
	T2	p63+	
	T3	p63-	
	T4	Vimentin+	
	Tx	Vimentin-	
	Tumor size	Cadherin+	
	N0	Cadherin-	
	N1/2	SATB+	
	Nx	SATB-	
	Lymph node number	GATA3+	
	Extent 1 organ	GATA-	
	Extent 2 organ	CKKL4-	
	Extent 3 organ	CKKL+	
	Extent 4 organ	MUC0AC+	
	Extent 5+ organ	MUC0AC-	
	V0		
	V1		
	L0		
	L1		
	Pn0		
	Pn1		
	R0		
	R1		
	Rx		

Supplement S5: *Lesion based features' permutation importance correlation triangle*

Supplement S6: *Patient based features' permutation importance correlation triangle*

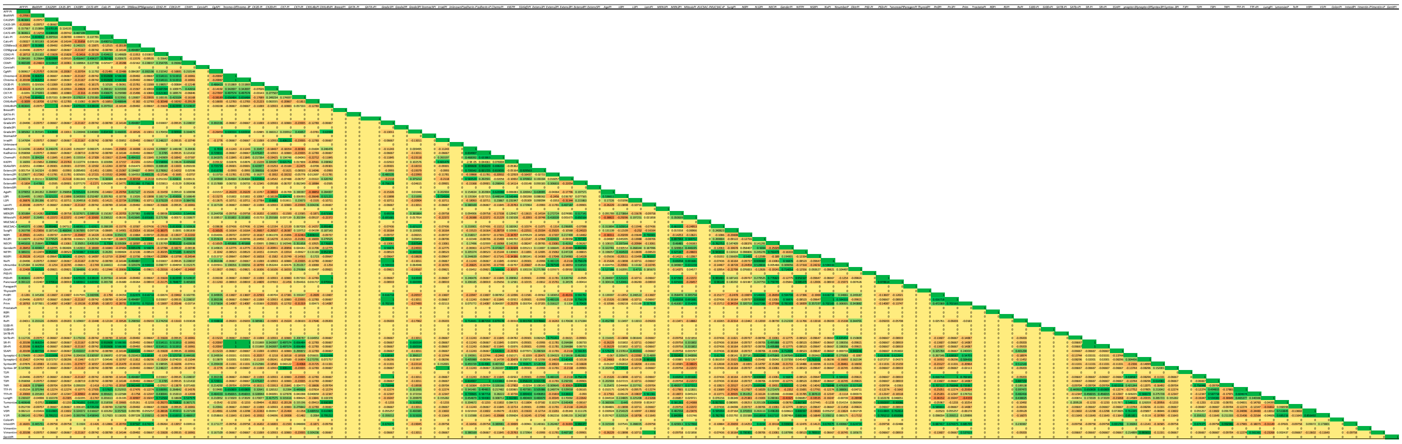

Supplement: Supplementary file 1 [file cancers-17-02935-s001.zip › Supplementary files.pdf]
